# Towards a standardized method of developing quality indicators for palliative care: protocol of the Quality indicators for Palliative Care (Q-PAC) study

**DOI:** 10.1186/1472-684X-12-6

**Published:** 2013-02-08

**Authors:** Kathleen Leemans, Joachim Cohen, Anneke L Francke, Robert Vander Stichele, Susanne JJ Claessen, Lieve Van den Block, Luc Deliens

**Affiliations:** 1 End-of-Life Care Research Group, Ghent University & Vrije Universiteit Brussel, Laarbeeklaan 103, Brussels, 1090, Belgium; 2 Department of Public and Occupational Health, EMGO Institute for Health and Care Research, VU University Medical Centre, Van der Boechorststraat 7, Amsterdam, 1081 BT, the Netherlands; 3 NIVEL. Netherlands Institute for health services research, Otterstraat 118-124, Utrecht, 3513 BN, the Netherlands; 4 Department of Family Medicine, Vrije Universiteit Brussel, Laarbeeklaan 103, Brussels, 1090, Belgium

**Keywords:** Quality indicators, Quality measurement, Palliative care, Quality of care, End of life care, Hospice care, Outcome measures, Developing method

## Abstract

**Background:**

In recent years, there have been several studies, using a wide variety of methods, aimed at developing quality indicators for palliative care. In this Quality Indicators for Palliative Care study (Q-PAC study) we have applied a scientifically rigorous method to develop a comprehensive and valid quality indicator set which can contribute to a standardized method for use in other countries.

**Methods and design:**

Firstly, an extensive literature review identified existing international quality indicators and relevant themes for measuring quality in palliative care. Secondly, the most relevant of these were selected by an expert panel. Thirdly, those prioritized by the experts were scored by a second multidisciplinary expert panel for usability and relevance, in keeping with the RAND/UCLA-method, combining evidence with consensus among stakeholders. This panel included carers and policymakers as well as patients and next-of-kin. Fourthly, the draft set was tested and evaluated in practice for usability and feasibility; the indicators were then translated into questionnaires presented to patients, next-of-kin and care providers. To encourage the acceptance and use of the indicators, stakeholders, including national palliative care organizations, were involved throughout the whole project.

**Conclusion:**

Our indicator development trajectory resulted in a set of quality indicators applicable to all patients in all palliative care settings. The set includes patient and relative perspectives and includes outcome, process and structure indicators. Our method can contribute internationally to a more standardized and rigorous approach to developing quality indicators for palliative care.

## Background

Quality of care is of interest to everyone receiving or providing palliative care [1]. Evaluation of quality informs care providers, administrators and policymakers about whether patients and their families are receiving care that fits their needs [2-4]. The challenge for quality improvement in palliative care is to develop effective ways for the quick and efficient assessment of service performance and outcomes, as this facilitates the modification of services and practices in order to improve the quality of care at individual and institutional levels [5-8]. For this purpose, quality indicators specifically for palliative care can be used to address issues unique to this type of care [9].

Although in the past decade a variety of studies have focused on quality indicators for palliative care, the methods found in the literature by which indicators were developed were not always clearly presented and not always seemed to follow a systematic approach involving different steps of the development process [10-26]. Two recent systematic efforts have developed a nationwide quality register system to measure outcomes and quality of palliative care services: the Palliative Care Outcomes Collaboration (PCOC)-study in Australia [27], and a similar registration project in Sweden [28]. While these projects have resulted in very useful national quality monitoring systems, they do not strictly make use of quality indicators but mainly focus on outcome measures. Additionally they did not seem to aim for a level of comprehensiveness necessary to evaluate the various dimensions of palliative care.

It appears that there is a need for a more standardized method of developing a comprehensive quality indicator set. Even at a more fundamental level, however, there is also a need for a common understanding of what quality indicators are. Quality indicators are well-defined and measurable aspects of care that give an indication of the quality of care delivered; they are generally expressed in a number or percentage, address a specific aspect of care or a related outcome, and are expressed at an aggregated level, often the level of care organisations [29-32]. They have to be clinically relevant, manageable and based on existing evidence, or consensus in the absence of such evidence [30]. Specific properties have to be described: a numerator (ie for which patients the indicator is positive) and a denominator (the group of patients being evaluated), a threshold value as a performance standard and exclusion criteria. Validated measurement instruments and relevant outcome measures are preferably used [33]. Confusion persists about outcome measures, measurement instruments and quality indicators, with these concepts often used interchangeably and mistakenly. Outcome measures are an essential component of quality, providing a way to evaluate patient- or family-level status and responses to treatment, measured on an individual level [34]. However, as quality indicators, they do not have standardized specifications detailing the eligible population, data collection procedures and types of analysis needed to calculate them [24] (see Table 1).

**Table 1:** Definitions and examples of outcome measures, quality indicators and quality measurement instruments

	**Outcome measure/ variable**	**Quality indicator**	**Quality measurement instrument**
**Definition**	An essential component of quality whereby the focus lies on patient’s or relatives outcomes, measured at an individual level.	Well defined and measurable aspect of care, generally expressed in a number or percentage, addressing a specific aspect of care or a related outcome. Quality indicators are usually expressed on aggregated level.	Instruments that can be used to monitor quality of care.
**Characteristics**	Concerns outcomes of care	Concerns structure, process or outcomes of care	Concerns structure, process or outcomes of care
	In palliative care, outcome measures provide a way to evaluate patient- and family-level status and response to treatment for symptoms and conditions in physical, psychological and other domains	Clinically relevant, manageable and based on existing evidence, or if not applicable on consensus	These instruments are used to measure outcome measures on individual level as well as quality indicators on aggregated level Individual and aggregated level
		Contains standardized specifications detailing the eligible population, data collection procedures and types of analyses needed to calculate the indicator	
	Individual level	Aggregated level	
**Example**	Pain intensity	Percentage of patients with moderate to severe pain [10]	Numerated rating scales (NRS)
	Quality of life Comfort	Extent to which patients indicate that caregivers respect their life stance [10]	McGill Quality of Life Questionnaire [44]
	Patient’s appraisal of the quality of care Relative’s appraisal of the quality of care	Extent to which direct relatives felt supported by the caregivers immediately after the patient’s death [10]	VOICES questionnaire [42,43]

Following the model proposed by Donabedian, quality indicators should be monitored in all three major areas of health care: structure, process and outcome [35]. Structure refers to the organization of care (eg presence of a written policy about visiting patients in the ICU), whereas process refers to the interaction between providers and receivers of care (eg psychosocial support being offered within the first 72 hours of admission to the ICU) [36]. Outcomes of care are at the level of the beneficiary of that care (eg adequate treatment of pain) [36].

With good examples of systematic and scientifically rigorous development trajectories for quality indicators for palliative care lacking in the literature, we intend this article to contribute to the establishment of a scientifically rigorous standardized and applicable method of measuring usable quality indicators. We do this by describing the protocol of a study developing a quality indicators set for palliative care for adults in Flanders (the Flemish speaking part of Belgium), applicable in all settings providing palliative care, thus providing an example usable by other countries interested in monitoring the quality of palliative care.

## Methods and design

**Figure 1:**
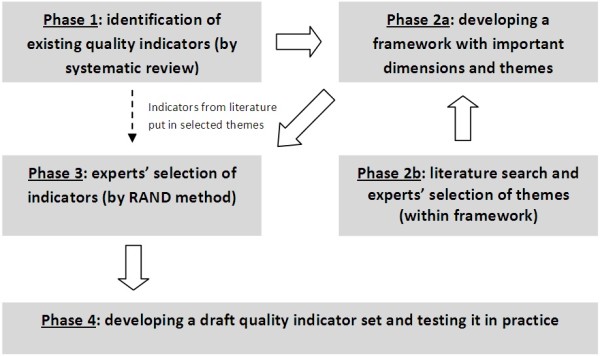
Flowchart of the 4 phases of the Flanders Study to develop Quality Indicators for Palliative Care.

In order to develop a comprehensive set of quality indicators to monitor the quality of palliative care in Flanders, four phases were followed in the development process: 1) identification of existing quality indicators, 2) development of a framework for quality of palliative care, 3) indicator selection by expert consultations and 4) testing the draft quality indicator set in palliative practice (Figure 1). The protocol of the present study was approved by the Ethical Review Board of Brussels University Hospital of the Vrije Universiteit Brussel.

### Phase 1: identification of existing quality indicators

To get an overview of previous attempts to develop quality indicators for palliative care, a thorough systematic literature review was performed, building on a review done in 2007 by Pasman et al [36]. With the field of quality indicators and the literature on quality measurements in palliative care developing quickly, it was important to update the existing review using the same method and search strategies but including the most recent literature published between December 2007 and May 2009.

As in the original review, we searched four international databases (Pubmed, PsychINFO, EMBASE and CINAHL). Two independent reviewers (KL and JC) followed the same procedures as in the original review by Pasman et al (results of this review update are integrated into a paper published in Journal of Pain and Symptom Management [37]).

### Phase 2: identifying domains and themes of quality palliative care

To achieve eventual comprehensiveness in the set of quality indicators we conceived a conceptual framework based on publications focussing on the determination of high quality palliative care consisting of several domains [38-40]: 1) physical, 2) psychological, social and existential, 3) information, communication, planning and decision making with patients, 4) with family and 5) with other carers, 6) type of care, 7) coordination and continuity, 8) support of friend or family carers and 9) structure of care (Table 2). Within these nine domains, we distinguished several themes (see Table 3 for an example of one domain with different themes).

**Table 2:** Selected domains for the quality of palliative care in Flanders

	**Domain**
1.	Physical treatment and care
2.	Psychological, social and existential treatment and care
3.	Information, communication, planning and decision making with the patient
4.	Information, communication, planning and decision making with the family
5.	Information, communication, planning and decision making with other caregivers
6.	Type of care at the end of life
7.	Coordination and continuity of care
8.	Support of family and informal caregivers
9.	Structure of care

**Table 3:** Example of themes within domains: Domain 1 ‘Physical treatment and care’

**Process of care**	
1.	To measure or evaluate general symptom burden
2.	To measure or evaluate pain
3.	To measure or evaluate nausea and problems with digestion
4.	To measure or evaluate fatigue and insomnia
5.	To measure or evaluate decubitus
6.	To measure or evaluate appetite
7.	To measure or evaluate problems with respiration/tightness of the chest
8.	To measure or evaluate delirium
9.	To measure or evaluate complaints on the mouth
10.	To treat or care for physical symptoms once observed with the patient
11.	Mentioning in the patient’s file the presence of symptoms
12.	Mentioning in the patient’s file the offered treatment and/or medication for the purpose of physical problems
13.	Mentioning in the patient’s file the result of the offered treatment and/or medication for the purpose of physical problems
**Outcome of care**	
1.	Low general symptom burden
2.	Absence of pain
3.	Absence of problems with respiration/ tightness of the chest
4.	Absence of delirium
5.	Absence of decubitus
6.	Absence of fatigue and insomnia
7.	Absence of complaints of the mouth
8.	Absence of nausea and problems with digestion
9.	A good appetite

In order to select the themes most important to quality palliative care we held a consultation of experts representing all relevant actors in palliative care, including patient and family perspectives. The panel consisted of nurses and other caregivers from all different specialized palliative care services, physicians from different specialities, coordinators of the Flemish Palliative Care networks, volunteers, spiritual and religous counsellors, bereaved family members and representatives of palliative patients i.e. large patient organisations such as the Flemish Cancer League and the Flemish Advisory Board for the Elderly.

The panel was asked to score each theme, as identified through the literature, on a scale from 1 (least important) to 5 (most important). Additionally we asked them to select the three most important themes per domain (questionnaires available upon request). Per domain themes were ranked from most important to least important based on the mean scores and the frequency with which the theme was indicated as most important within the domain. Based on the mean scoring (highest tertile across all domains) a number of themes were selected. To be selected, there had to be consensus among the panel members for each indicator, i.e. not more than four members scoring outside the mean range [1-9]. With every domain not then represented, we included one additional theme most often indicated as one of the three most important themes within the domains that were underrepresented. We choose to preserve the nine domains because the research team decided that all domains should be covered in order to have a comprehensive set. Additionally, experts were able to identify additional themes that did not come out of the literature review in a separate box for each domain and these were added if consensus among the experts was reached (i.e. more than one expert added this theme).

### Phase 3: indicator selection by expert consultation

The selected themes were then translated into quality indicators (i.e. with standardized specifications detailing the eligible population, data collection procedures, numerator and denominator), using the quality indicators found in the systematic literature review in the first phase. Then a second consecutive multidisciplinary palliative care expert panel was organized for another selection round. This panel, like the first panel, consisted of researchers in quality measurement or in palliative care, nurses and other caregivers in the field of palliative care, caregivers with a policy task in palliative care, bereaved family members and patient representatives. The experts could add quality indicators important to them not presented in the list and could discard those they found less important. For social and existential aspects of care, no indicators were found in the literature so experts were asked to suggest well-defined and measurable quality indicators for further development.

Unlike with the first expert consultation, we put together the second panel following the appropriateness method of the Research ANd Development corporation in collaboration with the University of California at Los Angeles (RAND/UCLA) [41]. This is the only systematic method for selecting quality indicators combining evidence with consensus among stakeholders. We chose to follow this method because, particularly for palliative care, quality indicators have to be developed using other evidence alongside expert opinion [29] as this area of health care has a limited or methodologically weak evidence base. Within this RAND/UCLA-method we executed the five prescribed steps [42]:

1. Comprehensive literature review on the topic (i.e. systematic literature review in phase 1) and recommendation of a preliminary set of quality indicators within the quality framework obtained through the first expert panel.

2. Recruiting expert clinicians from professional organisations reflecting the variety of specialities in palliative care and inviting them to join a panel for a two-stage process to rate the validity of the indicators. These experts included a representative from a patient organisation and a next-of-kin (eg partner, relative or friend most closely involved), as in the first panel round.

3. Sending the draft indicators by post to the panel members, who rated the indicators in terms of appropriateness and relevance as measures of quality on a scale from 1 (not appropriate or not necessary) to 9 (very appropriate or very necessary) (questionnaires available upon request).

4. Feeding back first-round scores to the panellists for a second round of scoring in a face-to-face panel meeting. Only indicators with a median score of 4, 5, 6 or higher but not reaching consensus were discussed, modified where necessary and rescored. The indicators with a median score of 7, 8 or 9 were automatically added to the quality indicator set and modified where necessary by the researchers.

5. Using the second-round scores to select only those indicators rated highly for validity and on which panel members had reached consensus. Indicators rated low or not reaching consensus among panellists were discarded.

After this second face-to-face consultation a draft set of quality indicators was produced (Figure 1).

### Phase 4: Developing and testing the draft quality indicator set in palliative practice

The next challenge was to test the draft indicator set in palliative care practice, in particular in order to assess its usability and feasibility (eg work load). Up until now, the draft set had been based on literature and expert opinion and hence needed operationalization and validation in practice, after which final adjustments could be made.

The indicators were transferred into questionnaires for patients, medical staff and next-of-kin (eg partner, relative or friend most closely involved) (Table 4). In order to obtain all information needed to calculate the selected indicators we composed our questionnaires using previously validated questions from existing questionnaires when these existing questions covered the quality indicator well. We for instance used questions of the Views Of Informal Carers – Evaluation of Services (VOICES) Questionnaire [43,44] and the McGill Quality of Life Questionnaire [45], particularly as these questionnaires are frequently used and already validated for a large group of respondents. For those indicators where no validated questions were available we composed questions ourselves. In a pre-test among patients, family members and caregivers, we tested this not yet validated questions and made an estimation of the time needed to complete each questionnaire.When patients were not able to complete the questionnaire themselves, a proxy was assigned.

**Table 4:** Distribution between observational units and study units during the testing phase

	**Observational unit**					
	**Patient**	**Proxy**	**Next-of-kin**	**Caregiver**		**Care institution**
**Study unit**	**before death**	**before death**	**after death**	**before death**	**after death**	**before death**
Care for the patient	x	x	x			
Care for the next of kin			x			
Process of care				x	x	
Structure of care						x

The questionnaires were divided over the observational units via four different palliative care services (representing all the different palliative care services in Flanders): the palliative home care team, the palliative care unit, the palliative support team in the hospital and the care home (only residents for whom a ‘palliative trajectory’ was initiated). A selection of services was recruited via the existing palliative care networks in Flanders: a call was sent out by the coordinators of these networks and services could volunteer to participate. After recruitment, a contact person for each service was appointed to organize the mailing of the questionnaires together with a researcher from the research team. The intention was to capture a cross section of the quality of palliative care in the different services at a particular moment, so one interrogation only was conducted. This demanded that a random selection was made of patients receiving palliative care services as well of those who had received palliative care services and had died between six weeks and six months earlier; a representative snapshot of the quality of existing care was thus created, taking into account coincidental patient mix confounders. With the quality of care susceptible to change over time, such a snapshot forms a basic starting point. The choice of a single measurement also makes the measurement of quality less burdensome for staff.

Four different types of questionnaire (available upon request), each measuring different quality indicators, were sent:

For adult patients currently receiving palliative care

For next-of-kin involved in the care of a patient who had died six weeks to six months previously

For the central formal care provider (a nurse in the palliative home care team and in the care home and a nurse or a physician in the palliative support team and the palliative care unit) about the care of a patient currently receiving palliative care

For general practitioner of patients who had died in the previous six weeks to six months

A maximum of 10 questionnaires was sent to any one nurse or physician.

We were able to list which patients, care providers and next-of-kin had already participated by the identifier numbers on the returned questionnaires attributed to each selected patient at the time of inclusion. In collaboration with the coordinator of each service involved, reminders were sent: one to patients and next-of-kin and up to two to care providers. As the researchers have not disposed of the list with patient, caregiver and family member contact details, identifying individuals through the identifier numbers on the questionnaires was not possible. After the reminders were sent and the questionnaires returned the collected data was analysed and quality indicators were calculated for each participating service.

The testing phase was then evaluated in terms of feasibility and usability. First telephone interviews with non-responding family members and family physicians were held to assess reasons for non-participation. This was done by a third independent party in order to preserve anonymity of patients and family members from the researchers. The contact person of the palliative service communicated the phone numbers of family and physicians directly to this independent person by email, based on the identifier numbers received by the researchers. Furthermore interviews were conducted with all coordinators and contact persons to evaluate the usability of the indicators and the procedure. Before the start of the interview all contact persons were asked to question the other caregivers about their experiences with the testing and the length of the questionnaire(s) they completed.

Based on the results of the test in terms of usability and feasibility, the indicator set, questionnaires and manual were adapted in order to be ready for use by the palliative services independently. We evaluated the instrument combining qualitative interviews with the caregivers involved about the workload and future use of the set and of quantitative psychometric analyses of the dataset gathered (e.g. number of missing answers per indicator). Furthermore, on request of the field, the indicator set was divided into a required minimal data set with complementary modules, each set usable separately or combined by the palliative services interested in quality measurement and improvement.

## Discussion

This article presents a method followed in Flanders (the Flemish speaking part of Belgium) for developing quality indicators for palliative care. The method comprises four phases: 1) a literature review identifying existing quality indicators, 2) a literature review and expert consultation to identify the important themes and domains for quality palliative care, 3) quality indicator selection by expert consultation following the internationally validated RAND method and 4) testing the draft quality indicator set in practice. This four-phase method combines the different phases from previous development processes in other countries (ie literature review, expert consultation, practical test) and additionally includes patient and next-of-kin perspectives, structure, process and outcome indicators and applicability to all patients in all palliative care services.

The method developed here can contribute to the development of a standardized and functional set of quality indicators for palliative care in other countries as it meets the standards of scientific rigour and the level of comprehensiveness required.

### Choosing a multi phased method leading to comprehensiveness

An important strength of the quality indicators development method described here is that it contains several phases to make the quality indicator set as comprehensive as possible, i.e. measuring quality of care for all adult palliative patients and across all domains relevant to palliative care. By combining a literature review on quality indicators with the building of a framework on important themes in palliative care, all aspects of palliative care have been taken into account. Previous sets of quality indicators for palliative care have tended to target specific populations such as cancer patients or vulnerable older people [9,16,18,46-48]. Our set, like that developed in the Netherlands is characterized by applicability to all setting and to all adult patient groups receiving palliative care [10] making it possible in the future, after assessing case mix adjusters, to compare scores of quality indicators not only over different disciplines but over different care settings.

### Choosing a combination of evidence through literature review and expert consultation

As the thinking about quality indicators and the literature about quality of palliative care is developing quickly, it is necessary to review the field regularly, especially when beginning to develop a new set of quality indicators. In the literature we found researchers developing quality indicators who had not performed such a review but had started from an existing quality indicator set such as Saliba et al. [22] who had selected indicators feasible for nursing home quality assessment from the original ACOVE quality indicator set. This raises the question of whether completely new indicators need to be developed for a particular country or whether existing indicators can be adjusted and validated in a set for that country [37]. Either way, a literature review can help to reveal existing indicators for further development and quality monitoring in any country.

Consultation of experts in palliative care in order to select the most important elements and indicators for measuring quality is a key element in the methodology described here and scoring by experts is important in assessing the validity and usefulness of the proposed indicators. Additionally it has the advantage that consultation of relevant palliative care actors enhances the acceptance and face-validity of the resulting indicator set among the palliative care community [10]. The composition of the panel should reflect all relevant actors within palliative care: those in medical practice and palliative care, policymakers, researchers, patient representatives and family. The latter two categories are often forgotten in quality indicator development but are essential to the creation of a comprehensive set of quality indicators reflecting all perspectives in palliative practice. The expert consultation can best be done by the RAND/UCLA-method [42].

### Choosing inclusion of outcome, process and structure indicators and involvement from all perspectives

Although measuring structure and process indicators from medical charts or administrative databases may be easier and cheaper, using only such sources for quality measurements may cause problems. Such databases can be limited, especially where indicators depend on precise documentation of issues such as communication, patient-reported outcomes or preferences [9]; such information can only be obtained from patients and their families themselves. On the other hand some researchers and care providers tend to see quality only through the perspective of the patient. This perspective does indeed provide the best indication of whether good quality care has been achieved [10], but data for calculating have to be derived from individual care users which, apart from the practical issues, also limits the possibility of measuring all relevant aspects of the quality of palliative care which might be susceptible to improvement. Therefore many authors [9,29,35,38,39,49] suggest that a quality indicator set, in order to be broad and comprehensive, must include structure and process as well as outcome indicators. We chose to translate our draft indicator set into questionnaires for the different respondent groups, patients, family and care providers, depending on which we considered would be the most reliable raters for selected quality indicators. Other quality researchers have based their evaluation of usability and feasibility on abstraction from patient records [11,14,23,25], thereby automatically limiting the comprehensiveness of an indicator set.

### Choosing a manageable testing of the quality indicator set

A crucial stage in the development process is the testing and evaluation of the set for usability and feasibility (eg work load) in practice; before it can be taken up on a nationwide scale the set needs operationalization and validation in palliative practice. Because of its extensive character, we felt it necessary to perform this testing phase in a small sample of palliative care service organisations.

To stimulate acceptance and use of quality indicators, stakeholders from palliative practice should be involved as early as possible in the development process. In Flanders we also collaborated closely with the Flemish Federation for Palliative Care, which helped to promote the project and enhance participation from care providers.

### Work in progress

The quality indicator set for palliative care for adults in Flanders is now fully developed and ready to be used in practice to monitor quality of palliative care. Before scores of quality indicators can be validly compared across different services and care providers, however, significant case mix adjusters and the discriminative power of the indicators need to be assessed. Only if future research in a larger sample confirms the discriminative power of the indicators can they be recommended for comparative information or benchmarking [10] while benchmarking itself can only be made possible by implementing the quality indicator set on a large scale in Flanders.

A standardized method for the development of quality indicators for palliative care can also offer opportunities on the international level; providing internationally comparable data [50].

## Conclusion

There is an increasing recognition of the need for quality indicators for palliative care in order to develop quality programmes across countries and to provide evidence to policymakers about the quality of the palliative care they are providing. However, a standardized method of developing quality indicators for palliative care has been lacking. In this paper we propose a method which meets the required level of scientific rigour and creates a sound basis for achieving the comprehensiveness needed in a set of quality indicators. The method suggested here combines standard literature analyses with multidisciplinary expert consultations involving all relevant actors within palliative care, following the RAND/UCLA-method, the only validated systematic method for quality indicator selection. Those wanting to develop indicators in other countries can use this standardized method to further develop and validate quality indicators for palliative care.

## Competing interests

The authors state that there is no conflict of interest.

## Author’s contributions

KL performed the study, participated in its design and drafted the manuscript. JC conceived of the study, participated in its design and coordination and helped to draft the manuscript. ALF and RVS participated in the design of the study and revised the article critically for important intellectual content. SJJC revised the article critically for important intellectual content. LVDB participated in the design of the study and revised the article critically for important intellectual content. LD conceived of the study, participated in its design and coordination and revised the article critically for important intellectual content. All authors read and approved the final manuscript.

## Pre-publication history

The pre-publication history for this paper can be accessed here:

http://www.biomedcentral.com/1472-684X/12/6/prepub

## References

[B1] Emanuel EJ Emanuel LL The promise of a good death Lancet 1998 351(2 SII21 SII29 9606363 10.1016/s0140-6736(98)90329-4

[B2] Singer PA Martin DK Kelner M Quality end-of-life care: patients' perspectives JAMA 1999 281 2 163 8 9917120 10.1001/jama.281.2.163

[B3] Steinhauser KE Clipp EC McNeilly M In search of a good death: observations of patients, families, and providers Ann Intern Med 2000 132 10 825 32 10819707 10.7326/0003-4819-132-10-200005160-00011

[B4] Teno JM Casey VA Welch LC Edgman-Levitan S Patient-focused, family-centered end-of-life medical care: views of the guidelines and bereaved family members J Pain Symptom Manage 2001 22 3 738 51 11532587 10.1016/s0885-3924(01)00335-9

[B5] Bowman KW Martin DK Singer PA Quality end-of-life care J Eval Clin Pract 2000 6 1 51 61 10807024 10.1046/j.1365-2753.2000.00232.x

[B6] Campbell SM Roland MO Buetow SA Defining quality of care Soc Sci Med 2000 51 11 1611 25 11072882 10.1016/s0277-9536(00)00057-5

[B7] Lorenz KA Lynn J Dy SM Evidence for improving palliative care at the end of life: a systematic review Ann Intern Med 2008 148 2 147 59 18195339 10.7326/0003-4819-148-2-200801150-00010

[B8] Rosenfeld K Wenger NS Measuring quality in end-of-life care Clin Geriatr Med 2000 16 2 387 400 10783435 10.1016/s0749-0690(05)70063-x

[B9] Seow H Snyder CF Mularski RA A framework for assessing quality indicators for cancer care at the end of life J Pain Symptom Manage 2009 38 6 903 12 19775860 10.1016/j.jpainsymman.2009.04.024

[B10] Claessen SJ Francke AL Belarbi HE A new set of quality indicators for palliative care: process and results of the development trajectory J Pain Symptom Manage 2011 42 2 169 82 21429703 10.1016/j.jpainsymman.2010.10.267

[B11] Dy SM Lorenz KA O'Neill SM Cancer Quality-ASSIST supportive oncology quality indicator set: feasibility, reliability, and validity testing Cancer 2010 116 13 3267 75 20564637 10.1002/cncr.25109

[B12] Earle CC Park ER Lai B Identifying potential indicators of the quality of end-of-life cancer care from administrative data J Clin Oncol 2003 21 6 1133 8 12637481 10.1200/JCO.2003.03.059

[B13] Earle CC Neville BA Landrum MB Evaluating claims-based indicators of the intensity of end-of-life cancer care Int J Qual Health Care 2005 17 6 505 9 15985505 10.1093/intqhc/mzi061

[B14] Grunfeld E Lethbridge L Dewar R Towards using administrative databases to measure population-based indicators of quality of end-of-life care: testing the methodology Palliat Med 2006 20 8 769 77 17148531 10.1177/0269216306072553 PMC3741158

[B15] Grunfeld E Urquhart R Mykhalovskiy E Toward population-based indicators of quality end-of-life care: testing stakeholder agreement Cancer 2008 112 10 2301 8 18361447 10.1002/cncr.23428 PMC3749155

[B16] Lorenz KA Rosenfeld K Wenger N Quality indicators for palliative and end-of-life care in vulnerable elders J Am Geriatr Soc 2007 55 Suppl 2 S318 S326 17910553 10.1111/j.1532-5415.2007.01338.x

[B17] Lorenz KA Dy SM Naeim A Quality measures for supportive cancer care: the Cancer Quality-ASSIST Project J Pain Symptom Manage 2009 37 6 943 64 19359135 10.1016/j.jpainsymman.2008.05.018

[B18] Miyashita M Morita T Ichikawa T Quality indicators of end-of-life cancer care from the bereaved family members' perspective in Japan J Pain Symptom Manage 2009 37 6 1019 26 19321296 10.1016/j.jpainsymman.2008.05.015

[B19] Mularski RA Curtis JR Billings JA Proposed quality measures for palliative care in the critically ill: a consensus from the Robert Wood Johnson Foundation Critical Care Workgroup Crit Care Med 2006 34 11 S404 S411 17057606 10.1097/01.CCM.0000242910.00801.53

[B20] National Quality Forum National voluntary consensus standards for symptom management and end-of-life care in cancer patients 2006 Washington DC: National Quality Forum

[B21] Pastrana T Radbruch L Nauck F Outcome indicators in palliative care-how to assess quality and success, Focus group and nominal group technique in Germany Support Care Cancer 2009 18 7 859 868 19701782 10.1007/s00520-009-0721-4 PMC3128732

[B22] Saliba D Solomon D Rubenstein L Feasibility of quality indicators for the management of geriatric syndromes in nursing home residents J Am Med Dir Assoc 2004 5 5 310 9 15357888

[B23] Sato K Miyashita M Morita T Reliability assessment and findings of a newly developed quality measurement instrument: quality indicators of end-of-life cancer care from medical chart review at a Japanese regional cancer center J Palliat Med 2008 11 5 729 37 18588405 10.1089/jpm.2007.0227

[B24] Schenck AP Rokoske FS Durham DD Cagle JG Hanson LC The PEACE Project: identification of quality measures for hospice and palliative care J Palliat Med 2010 13 12 1451 9 21155640 10.1089/jpm.2010.0238

[B25] Twaddle ML Maxwell TL Cassel JB Palliative Care Benchmarks from Academic Medical Centers J Palliat Med 2007 10 1 86 98 17298257 10.1089/jpm.2006.0048

[B26] Yabroff KR Mandelblatt JS Ingham J The quality of medical care at the end-of-life in the USA: existing barriers and examples of process and outcome measures Palliat Med 2004 18 3 202 16 15198133 10.1191/0269216304pm880oa

[B27] Eagar K Watters P Currow DC Aoun SM Yates P The Australian Palliative Care Outcomes Collaboration (PCOC) measuring the quality and outcomes of palliative care on a routine basis Aust Health Rev 2010 34 2 186 92 20497731 10.1071/AH08718

[B28] Lundstrom S Axelsson B Heedman PA Fransson G Furst CJ Developing a national quality register in end-of-life care: The Swedish experience Palliat Med 2012 26 4 313 21 21737480 10.1177/0269216311414758

[B29] Campbell SM Braspenning J Hutchinson A Marshall MN Research methods used in developing and applying quality indicators in primary care BMJ 2003 326 7393 816 9 12689983 10.1136/bmj.326.7393.816 PMC1125721

[B30] Ostgathe C Voltz R Quality indicators in end-of-life care Curr Opin Support Palliat Care 2010 4 3 170 3 20489644 10.1097/SPC.0b013e32833add10

[B31] Shekelle PG Maclean CH Morton SC Wenger NS Assessing care of vulnerable elders: methods for developing quality indicators Ann Intern Med 2001 135 8 647 52 Pt 2 11601947 10.7326/0003-4819-135-8_part_2-200110161-00003

[B32] Singer PA Martin DK Bowman K Quality end-of-life care: where do we go from here? J Palliat Med 2000 3 4 403 5 15859691 10.1089/jpm.2000.3.4.403

[B33] Bausewein C Simon ST Benalia H Implementing patient reported outcome measures (PROMs) in palliative care users' cry for help Health Qual Life Outcomes 2011 9 27 21507232 10.1186/1477-7525-9-27 PMC3112059

[B34] Mularski RA Dy SM Shugarman LR A systematic review of measures of end-of-life care and its outcomes Health Serv Res 2007 42 5 1848 70 17850523 10.1111/j.1475-6773.2007.00721.x PMC2254566

[B35] Donabedian A Quality assurance: structure, process and outcome Nurs Stand 1992 7 11 4 5 1489693

[B36] Pasman HR Brandt HE Deliens L Francke AL Quality indicators for palliative care: a systematic review J Pain Symptom Manage 2009 38 1 145 56 19615636 10.1016/j.jpainsymman.2008.07.008

[B37] De Roo M Leemans K Claessen SJ Quality indicators for palliative care: update of a systematic review J Pain Symptom Manage 2013 In press 10.1016/j.jpainsymman.2012.09.013 23809769

[B38] Ferrell F Overview of the domains of variables relevant to end-of-life care J Palliat Med 2005 8 22 9 10.1089/jpm.2005.8.s-22 16499465

[B39] Stewart AL Teno J Patrick DL Lynn J The concept of quality of life of dying persons in the context of health care J Pain Symptom Manage 1999 17 2 93 108 10069149 10.1016/s0885-3924(98)00131-6

[B40] Teno JM Clarridge B Casey V Edgman-Levitan S Fowler J Validation of Toolkit After-Death Bereaved Family Member Interview J Pain Symptom Manage 2001 22 3 752 8 11532588 10.1016/s0885-3924(01)00331-1

[B41] Fitch K Bernstein S Aguilar M Burnand B LaCalle J Lazaro P The RAND/UCLA Appropriateness Method User's Manual 2001 Santa Monica, CA: RAND Corporation

[B42] van der Ploeg E Depla MF Shekelle P Rigter H Mackenbach JP Developing quality indicators for general practice care for vulnerable elders; transfer from US to The Netherlands Qual Saf Health Care 2008 17 4 291 5 18678728 10.1136/qshc.2007.023226

[B43] Addington-Hall J VOICES (Vieuws of INformal Carers - Evaluation of Services 1998 London: King's College School of Medicine and Dentistry

[B44] Addington-Hall J Karlsen S Care for the Dying in Cornwall: A Survey of Bereaved Relatives. Final Report 1999 London: Department of Palliative Care and Policy

[B45] Cohen SR Mount BM Bruera E Validity of the McGill Quality of Life Questionnaire in the palliative care setting: a multi-centre Canadian study demonstrating the importance of the existential domain Palliat Med 1997 11 1 3 20 9068681 10.1177/026921639701100102

[B46] Lorenz KA Lynn J Dy S Quality measures for symptoms and advance care planning in cancer: a systematic review J Clin Oncol 2006 24 30 4933 8 17050878 10.1200/JCO.2006.06.8650

[B47] Seow H Snyder CF Shugarman LR Developing quality indicators for cancer end-of-life care: proceedings from a national symposium Cancer 2009 115 17 3820 9 19514090 10.1002/cncr.24439

[B48] Wenger NS Rosenfeld K Quality indicators for end-of-life care in vulnerable elders Ann Intern Med 2001 135 8 677 85 Pt 2 11601950 10.7326/0003-4819-135-8_part_2-200110161-00006

[B49] Donaldson MS Field M Measuring quality of care at the end of life Arch Intern Med 1998 158 2 121 8 9448550 10.1001/archinte.158.2.121

[B50] Casarett DJ Teno J Higginson I How Should Nations Measure the Quality of End- of-Life Care for Older Adults? Recommendations for an International Minimum Data Set J Am Geriatr Soc 2006 54 11 1765 71 17087706 10.1111/j.1532-5415.2006.00925.x

